# Sports Facility Use and Perceptions of Exercise Effectiveness: A Nationwide Survey of People with Disabilities in South Korea

**DOI:** 10.3390/healthcare13040399

**Published:** 2025-02-12

**Authors:** Kyung-Hun Cho, Jae Seung Chang

**Affiliations:** 1 Department of Physical Education, Kyung Hee University, Yongin 17104, Republic of Korea; bangbum119@khu.ac.kr; 2 Department of Sports Science, College of Life Science and Nano Technology, Hannam University, Daejeon 34430, Republic of Korea

**Keywords:** disabled people, physical activity, exercise, sports facility, perception of exercise effectiveness

## Abstract

**Objectives:** This study examined how the benefits of exercise vary across different dimensions, such as physical health, psychological well-being, and social interaction, for individuals with disabilities, focusing on their use of sports facilities. **Methods:** Based on the 2019 National Survey on Sports Participation among People with Disabilities in South Korea, 3726 participants were analyzed using stratified chi-square tests, one-way analysis of variance, analysis of covariance, and logistic regression methods, adjusting for covariates as appropriate. **Results:** This study’s results indicate that sports facility users showed higher positive perceptions of exercise benefits compared to non-users. Notable differences in the perceived effectiveness of exercise benefits were observed in physical health and fitness (odds ratio (OR), 1.31; 95% CI, 1.07–1.62), stress relief and psychological stability (OR, 1.38; 95% CI, 1.11–1.71), daily vitality and motivation (OR, 1.62; 95% CI, 1.31–2.00), and a general sense of happiness from exercise participation (OR, 1.32; 95% CI, 1.12–1.57), whereas the perception of medical cost savings did not vary (OR, 1.08; 95% CI, 0.93–1.27). **Conclusions:** These findings underscore the importance of accessible sports facilities and the promotion of their active use to improve the quality of life for individuals with disabilities. Political and practical initiatives are essential for improving both physical and mental well-being among individuals with disabilities.

## 1. Introduction

Individuals with disabilities experience higher rates of chronic diseases and limited access to preventive care, emphasizing the need for targeted public health interventions [1]. Due to their lower physical activity levels, they are more vulnerable to health deterioration caused by overweight and obesity, which subsequently increase the risk of chronic metabolic diseases such as hypertension, diabetes, and dyslipidemia [2,3]. Consequently, the risk of secondary conditions such as coronary artery disease, stroke, and cancer is also elevated [3]. Therefore, enhancing physical activity and improving access to healthcare and rehabilitation facilities for people with disabilities have emerged as critical goals.

Perceptions of the efficacy and benefits of exercise are crucial factors in recognizing the importance of physical activity and determining willingness and actual participation in such activities [4]. The quality and quantity of physical activity are commonly classified by intensity and total amount, with moderate to vigorous exercises distinguished from low-intensity activities, leading to various psychological and physiological adaptations [5,6]. Moderate to vigorous exercise brings numerous positive effects, such as improvements in cardiorespiratory fitness, metabolic function, muscle mass and strength, and mental health [6,7]. However, individuals with disabilities often fail to fully attain these benefits due to their lower rates of participation in physical activities compared to non-disabled individuals [8].

This issue is further exacerbated by the limited availability of exercise programs and facilities adapted to meet the needs of individuals with disabilities. People with disabilities frequently face barriers such as limited access to suitable exercise facilities, high costs, and a lack of qualified instructors, which may present challenges to their participation in regular physical activity and exercise [9]. Meanwhile, social prejudice and lack of awareness greatly impede their participation in physical activities; many are hesitant to engage in exercise because of negative societal perceptions toward their physical vulnerabilities and limitations [10]. In South Korea, cultural norms and societal perceptions further exacerbate these challenges. Traditional views emphasizing physical limitations over capabilities contribute to stigma and restrict opportunities for physical activity [11]. Economic factors, such as insufficient funding for accessible sports facilities and high participation costs, further limit access [12]. Despite recent initiatives aimed at improving accessibility and awareness, barriers to participation remain significant [13]. These challenges are not unique to South Korea. Globally, individuals with disabilities are 16–62% less likely to meet physical activity guidelines and are at a higher risk of serious health problems related to inactivity compared to people without disabilities [14]. This global perspective highlights the widespread nature of these issues and underscores the importance of addressing them through context-specific and internationally informed strategies.

The general benefits of physical activity for individuals with disabilities have been well documented in previous research [15,16]. However, studies exploring how perceptions of exercise benefits influence the actual utilization of sports facilities among this population are notably scarce. This gap should be addressed to develop interventions that promote physical activity while improving the perceived value and accessibility of such activities for individuals with disabilities. Therefore, the present study aimed to investigate the perceptions of exercise benefits among individuals with disabilities by assessing their use and frequency of use of sports facilities based on data from the national survey on sports participation. This study’s findings may contribute to the development of effective policies and practical strategies that enhance physical activity among individuals with disabilities and provide foundational data that support an improvement in health outcomes and the evaluation of sports participation policies for this population.

## 2. Materials and Methods

### 2.1. Study Population

**Figure 1:**
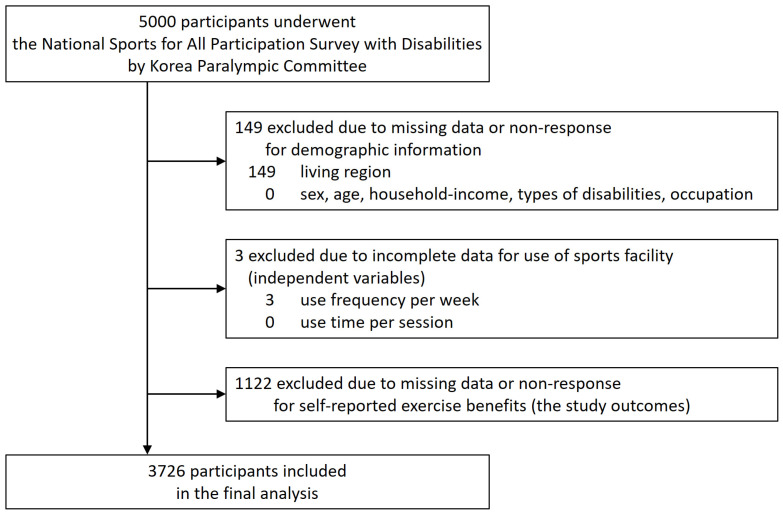
Flowchart of study’s participant selection process.

This study utilized the dataset from the 2019 National Survey on Sports Participation among People with Disabilities in South Korea. The data were collected from 5000 individuals (aged 10 to 69 years) who registered as disabled with the government. The sampling process was stratified by type and severity of disability, as well as by region, through systematic selection. This stratified sampling method ensured that the dataset represented the general population of individuals with disabilities in South Korea by providing proportional representation across the 17 provinces. The sample size of 5000 was determined based on a power analysis to achieve a 95% confidence level with a margin of error of ±1.38%, ensuring methodological rigor. For this study, individuals who did not report self-perceived exercise benefits (*n* = 1122), lacked demographic information (*n* = 149), and had incomplete sports facility usage data (*n* = 3) were excluded. Finally, 3726 individuals were analyzed (Figure 1).

### 2.2. Data Collection

The data were collected using a structured questionnaire, through telephone interviews, household visits, and online surveys. The interviewers conducting the surveys received prior training to ensure that they were familiar with the questionnaire and ensure effective communication with respondents. The questionnaire was administered through a face-to-face interview, allowing respondents to complete it themselves. The interviewers provided support in completing the questionnaire when needed. To minimize potential biases related to self-reported data, the survey used clear and concise questions reviewed by experts to ensure reliability. The respondents were provided with examples and clarifications to avoid misunderstandings. Additionally, the face-to-face format of the interviews enabled the interviewers to address ambiguities and verify the responses in real time. The 2019 National Survey on Sports Participation among People with Disabilities was conducted independently, without any involvement from the researchers in this study. The dataset, sourced from publicly available government records, was analyzed to address a gap in the existing literature regarding perceptions of exercise benefits among individuals with disabilities. The questionnaire used in the survey assessed perceptions of exercise benefits across several domains, including improvements in health and physical fitness, stress relief and psychological stability, daily life functionality (e.g., work, school, and family life), and reductions in medical costs. One example question was as follows: “Do you think exercise helps improve your health and physical fitness?”. Respondents were asked to rate their perceptions on a 5-point Likert scale, with the options including: “Not effective at all”, “Not effective”, “Somewhat effective”, “Effective”, and “Very effective”. The questionnaire was validated through expert reviews and pilot testing, which confirmed its clarity and relevance for the target population.

### 2.3. Study Design and Measures

This cross-sectional study aimed to investigate the association between perceptions of exercise’s efficacy and benefits and the use of sports facilities among individuals with disabilities. The data were collected through self-reported questionnaires, which contained components on demographic characteristics, sports facility usage, and perceptions regarding exercise efficacy and benefits. Demographic characteristics such as gender, age, residential area, type and severity of disability, occupation, and household income were collected. Certain variables, such as gender, income, and type of disability, were included as covariates in the statistical analyses because of their known influence on physical activity participation. These variables were chosen based on prior research indicating their role as potential confounders in understanding exercise behaviors. Perceptions of exercise benefits were assessed across five main domains: improvements in health and physical fitness, stress relief and psychological stability, vitality and motivation in daily life, reduction in medical costs, and overall happiness.

### 2.4. Data Analysis

All of the analyses were performed using SPSS version 26.0 (IBM Corp., Armonk, NY, USA), with statistical significance set at *p* < 0.05 (two-tailed). Data visualization was conducted using GraphPad Prism version 7 (GraphPad Software, San Diego, CA, USA). Descriptive statistics were applied to describe the demographic, behavioral, and perceptual characteristics of the study participants. Stratified chi-square tests were conducted to examine differences in the perceptions of exercise efficacy and benefits between users and non-users of sports facilities. Subgroup analyses based on the frequency of sports facility use were carried out using analysis of variance (ANOVA) and analysis of covariance (ANCOVA). Participants were divided into four groups based on how often they used sports facilities: non-users (NU), 1–2 times per week (T1), 3–4 times per week (T2), and 5 or more times per week (T3). Stepwise logistic regression analyses were employed to evaluate the association between sports facility usage and perceptions of exercise efficacy and benefits, with the odds ratios (ORs) calculated at each step with and without adjustments for potential covariates. 

## 3. Results

Of the 3726 participants included in the final analysis, 61.6% were men (*n* = 2295) and 38.4% were women (*n* = 1431). The age distribution was as follows: 10–19 years (10.3%), 20–29 years (14.6%), 30–39 years (19.5%), 40–49 years (22.3%), 50–59 years (22.8%), and 60–69 years (10.5%). By type of disability, the most common category was physical disabilities at 35.1% (*n* = 1306), followed by hearing disabilities at 16.2% (*n* = 604), intellectual disabilities at 15.6% (*n* = 581), brain lesions at 13.9% (*n* = 517), visual disabilities at 13.5% (*n* = 503), and other disabilities at 5.7% (*n* = 215). The occupational distribution was unemployed (49.5%), homemakers (17.5%), self-employed/service workers (11%), construction/manufacturing workers (6%), office/administrative staff (4.4%), agricultural/fishery workers (4%), students (3.9%), other occupations (3.1%), and managers/supervisors (0.6%) (Table 1). Regarding sports facility usage, 62.4% of the respondents (*n* = 2324) were users, while 37.6% (*n* = 1402) were non-users. Among the users, the weekly frequency of use was 1–2 times for 40.3% (*n* = 936), 3–4 times for 34.2% (*n* = 793), and 5 or more times for 25.5% (*n* = 595).

**Figure 2:**
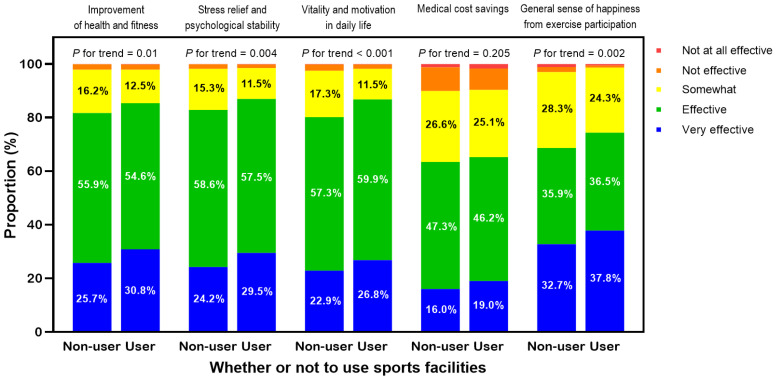
Stacked bar chart representing subjective perceptions of exercise benefits according to sports facility usage among individuals with disabilities. *P* for trends were calculated using stratified chi-square tests.

Figure 2 shows notable differences in perceived exercise benefits between users and non-users of sports facilities. In the domain of health and fitness improvement, the proportion of sports facility users who reported that exercise was “effective or very effective” was 3.8% higher (85.4%) than that of non-users (81.6%) (*p* for trend < 0.05). For daily vitality and motivation, users showed a 4.2% higher response rate (87.0%) compared to non-users (82.8%) (*p* for trend < 0.001). In stress relief and psychological stability, users showed a 6.5% higher response rate (86.7%) compared to non-users (80.2%) (*p* for trend < 0.01). Regarding overall happiness, the response rate for users was 5.7% higher (74.3%) than that of non-users (68.6%) (*p* for trend < 0.01). In terms of medical cost savings, no significant difference was found between users and non-users (Figure 2). The greatest difference was observed in the domain of stress relief and psychological stability, followed by daily vitality and motivation, overall happiness, and health and fitness improvement.

**Figure 3:**
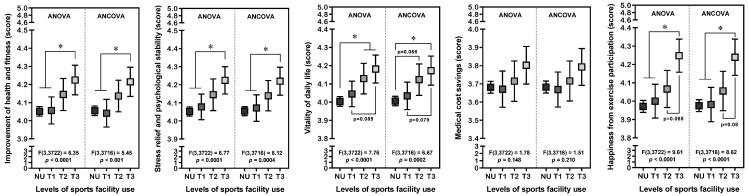
A comparison of subjective perception scores for exercise effectiveness according to the levels of sports facility use. The data were analyzed using analysis of variance (ANOVA) and analysis of covariance (ANCOVA) with Bonferroni’s correction applied for post hoc comparisons. Covariates included sex, age (10-year intervals), types of disabilities, occupation, living regions, and household income. *p*-values are provided to indicate significance. * *p* < 0.05.

Figure 3 presents the differences in perceived exercise benefits according to the frequency of sports facility use. A higher frequency of sports facility use was associated with a progressive increase in the mean scores for most domains of perceived exercise benefits (all *p* for trend < 0.0001), except for medical cost savings. According to post hoc analyses, the user group with the highest frequency (T3) in the domain of health and fitness improvement indicated a mean score of 4.224, which was significantly higher than that of the non-user group (NU) and the lowest-frequency user group (T1), whose mean scores were 4.051 and 4.056, respectively (both *p* < 0.05). Similar results were observed for stress relief and psychological stability, where the T3 group showed higher scores compared to the NU and T1 groups [4.224 (T3) vs. 4.052 (NU) and 4.077 (T1); both *p* < 0.05]. In terms of daily vitality and motivation, the T2 and T3 groups showed mean scores of 4.129 and 4.182, respectively, which are higher than the mean score of 4.003 for the NU group (both *p* < 0.05). For overall happiness, the T3 group indicated a mean score of 4.248, which is higher than the scores of the NU and T1 groups, 3.971 and 4.001, respectively (both *p* < 0.05). No significant differences were observed among the groups in the scores for medical cost savings (*p* for trend > 0.05). The differences among the groups remained consistent in the ANCOVA with Bonferroni’s correction, adjusting for confounding variables such as gender, age, type of disability, occupation, residential area, and household income (Figure 3). The most notable difference was observed in the domain of overall happiness, followed by enhancements in stress relief and psychological stability, daily vitality and motivation, and health and fitness.

Table 2 presents the odds ratios (ORs) for positive perceptions of exercise effectiveness based on sports facility usage, including both crude and stepwise-adjusted models for potential covariates (Models 1, 2, and 3). Logistic regression analysis showed that individuals who used sports facilities were more likely to report positive perceptions of exercise benefits than non-users, as indicated by higher odds ratios (ORs). For health and fitness improvement, the crude model indicated that facility users had approximately 31% higher odds of perceiving exercise as beneficial compared to non-users (OR, 1.31; 95% CI, 1.07–1.62; *p* < 0.05). In the fully adjusted model (Model 3), this association remained significant, with users having 25% higher odds of positive perceptions (OR, 1.25; 95% CI, 1.01–1.54; *p* < 0.05). For stress relief and psychological stability, the crude model showed that facility users had around 38% higher odds of reporting positive perceptions (OR, 1.38; 95% CI, 1.11–1.71; *p* < 0.01). After full adjustment, the odds remained 34% higher for positive perceptions (OR, 1.34; 95% CI, 1.07–1.67; *p* < 0.05). Regarding daily vitality and motivation, the crude model indicated 62% greater odds of positive perceptions among users (OR, 1.62; 95% CI, 1.31–2.00; *p* < 0.001), which was adjusted to 54% in Model 3 (OR, 1.54; 95% CI, 1.24–1.91; *p* < 0.001). For overall happiness, sports facility users in the crude model showed 32% higher odds of perceiving exercise positively (OR, 1.32; 95% CI, 1.12–1.57; *p* < 0.001), with significance retained at 23% higher odds in the fully adjusted model (OR, 1.23; 95% CI, 1.04–1.47; *p* < 0.05). No significant differences in ORs were observed for perceptions of medical cost savings in either the crude or adjusted models.

## 4. Discussion

This study examined the relationship between exercise-related behavior, particularly the frequency of sports facility use, and perceptions of exercise efficacy and benefits among individuals with disabilities. The findings demonstrate that a higher frequency of sports facility use is associated with more positive perceptions across various aspects of health and well-being. This suggests that the regular use of sports facilities enhances both physical and mental health for individuals with disabilities, thereby contributing to economic benefits and an overall improvement in quality of life [1,3,9]. Individuals with disabilities who regularly utilized sports facilities reported greater benefits in each domain, such as health and fitness improvement, stress relief and psychological stability, daily vitality and motivation, and overall happiness compared to those who did not use such facilities. These results underscore the role of sports facilities in promoting physical activity among individuals with disabilities, which leads to positive experiences across these domains [17]. Significant differences were particularly evident in health and fitness improvement, as well as daily vitality and motivation, indicating that the active and voluntary use of sports facilities contributes not only to physical health but also to mental well-being [18]. These findings are consistent with previous research showing that exercise participation in individuals with disabilities significantly improves psychological well-being, including reductions in anxiety and depressive symptoms while enhancing self-esteem and social connectedness [19,20]. Moreover, such benefits are often amplified when exercise is conducted in group settings, which provide additional opportunities for social interaction and peer support [21].

The domain-specific analysis of quality-of-life-related perceptions is a notable strength of this study. This study provides a more comprehensive examination, emphasizing the broader importance of physical activity for overall quality of life [20,22,23], while previous research primarily focused on the health improvements associated with physical activity among individuals with disabilities [15,16,24]. Thus, our findings suggest that the broader impacts of physical activity that encompass not just physical health but also psychological well-being, social interaction, and overall life satisfaction are critical to consider when developing policies and programs aimed at enhancing the quality of life for individuals with disabilities. These insights are relevant not only in South Korea but also in other countries where individuals with disabilities face similar barriers to accessing sports facilities. Globally, strategies to improve access to inclusive and adaptive sports facilities could draw on the findings of this study to inform best practices and policy recommendations [25].

The subgroup analysis based on the frequency of sports facility use revealed significant differences in perceptions of exercise benefits. Participants who used sports facilities more frequently, particularly those with accessible and appropriate accommodations tailored to the needs of individuals with disabilities, reported higher scores for positive perceptions of health and fitness improvement, stress relief and psychological stability, daily vitality, and overall happiness. These findings suggest that the accessibility and suitability of facilities tailored to individuals with disabilities are essential for increasing the frequency of their participation in physical activity, thereby enhancing their overall well-being [26,27,28]. As the results of the present study indicate, the usage of sports facilities may positively influence mental health. Social interactions and support gained through exercise are key contributors to psychological stability among individuals with disabilities [27,29]. Numerous studies have demonstrated that physical activity plays a pivotal role in reducing depression and anxiety while simultaneously enhancing self-esteem [30]. These results further underscore the importance of exercise in achieving psychological stability and fostering social connections within this population [29,31,32].

In the present study, we did not observe significant differences in the perception of medical cost savings associated with the use of sports facilities. This may be attributed to the fact that individuals with disabilities generally incur higher fixed medical expenses compared to non-disabled individuals, making it challenging for them to perceive substantial economic benefits solely from using sports facilities [33]. This unexpected finding warrants further exploration in future research. Studies could investigate whether factors such as the affordability of facilities, geographic accessibility, or government subsidies impact perceptions of cost savings in this population. Additionally, exploring whether different types of disabilities influence perceptions of economic benefits could provide deeper insights [34,35]. Although some public welfare facilities supported by the government and community offer services at low costs, these services are often insufficient. Moreover, the majority of sports facilities operate on a market-based model, which requires users to pay for their services [29]. Previous studies have also emphasized that cost is a significant concern for individuals with disabilities who wish to travel or engage in various activities [28,29,36]. Such economic burdens indicate that people with disabilities face greater barriers compared to non-disabled individuals when it comes to spending on leisure and physical activities [37]. Nonetheless, accumulating research demonstrates that consistent physical activity and exercise participation can effectively prevent and alleviate chronic and secondary health conditions, resulting in long-term medical cost savings [33]. Given this evidence, reducing opportunity costs and lowering barriers to entry for physical activity and exercise participation is a crucial strategy for alleviating the socioeconomic burden of healthcare costs for people with disabilities [31]. Therefore, implementing interventions at institutional and community levels, along with organized public policies, is essential to mitigating financial barriers for this population and promoting their active participation in physical activity and exercise [8].

The implications of this study provide several policy and practical recommendations for enhancing physical activity among individuals with disabilities. First, there is a critical need to expand and improve access to dedicated exercise facilities for this population. Second, the development and support of tailored exercise programs are crucial. Third, campaigns and promotional activities that encourage physical activity participation should be implemented. Finally, collaboration and support from families and communities are essential. Enhancing physical activity among individuals with disabilities could noticeably improve their health and well-being, suggesting that it should be a priority in public health policy [29,37]. Expanding exercise facilities and programs tailored to the specific needs of individuals with disabilities can augment the health benefits derived from physical activity [38]. Ultimately, these efforts may improve the management and prevention of chronic diseases among vulnerable populations, leading to an enhanced overall quality of life for individuals with disabilities [9,14,39].

This study has several limitations. One is the reliance on self-reported data, which may introduce biases such as the over- or underestimation of exercise perceptions and facility usage. Incorporating objective measures or observational methods in future research could help complement self-reported findings and enhance data accuracy. Another limitation is the absence of classifications on disability severity. Studies that include such detailed information could offer more comprehensive and meaningful insights into exercise perceptions across varying levels of disability. Additionally, the use of cross-sectional data limits the ability to establish causality between perceptions of exercise benefits and sports facility usage. While this study provides valuable associations, it cannot determine the directionality of the relationships observed. Future research could address this limitation by employing longitudinal study designs to track changes over time and explore causal pathways more effectively.

## 5. Conclusions

This study underscores the critical role of physical activity in improving the health and well-being of individuals with disabilities. Participation in physical activity contributes to enhanced physical health, mental stability, social interactions, and economic benefits. These findings align with previous studies highlighting the benefits of physical activity for both mental and physical health, further reinforcing the importance of accessible sports facilities. To ensure the practical implementation of these findings, governments, policymakers, and community organizations should collaborate to develop accessible infrastructure and tailored programs that meet the specific needs of individuals with disabilities. Strengthening support systems through collaboration with families and communities can encourage the utilization of sports facilities, thereby optimizing these outcomes. Moreover, these findings have global implications, serving as a foundation for establishing inclusive practices and policies that enhance access to physical activity for marginalized populations worldwide. An integrated approach that combines infrastructure development, tailored program design, economic support, and increased social awareness may be key to improving the overall quality of life for the disabled population. Continued research is required to explore the long-term impacts of accessibility interventions and to investigate specific strategies for addressing social stigma and economic constraints. Future studies should also examine how tailored policies, such as financial incentives for inclusive facilities and targeted public awareness campaigns, can further enhance participation.

## Figures and Tables

**Table 1:** Demographic characteristics of study population.

Variables	Categories	Frequency (N)	Proportion (%)
Sex	Men	2295	61.6
	Women	1431	38.4
Age	10–19	103	2.8
	20–29	252	6.8
	30–39	249	6.7
	40–49	499	13.4
	50–59	900	24.2
	60–69	1723	46.2
Household income (KRW million)	<1	2902	77.9
	1–2	496	13.3
	2–3	187	5
	3–4	74	2
	4–5	24	0.6
	≥5	43	1.2
Region	Seoul	600	16.1
	Incheon/Gyeonggi	1080	29
	Daejeon/Chungcheong	537	14.4
	Gwangju/Jeolla	467	12.5
	Daegu/Gyeongbuk	230	6.2
	Busan/Ulsan/Gyeongnam	611	16.4
	Gangwon/Jeju	201	5.4
Types of disabilities	Physical disability	1277	34.3
	Visual impairment	563	15.1
	Hearing/speech impairment	519	13.9
	Intellectual/autistic disorder	638	17.1
	Brain lesion disorder	544	14.6
	Other disabilities	185	5
Occupation	Manager/supervisor	22	0.6
	Office/administrative position	164	4.4
	Self-employed/service worker	409	11
	Construction/production worker	224	6
	Agriculture/fishery	148	4
	Student	146	3.9
	Housewife/husband	652	17.5
	Unemployed	1846	49.5
	Other occupations	115	3.1
Use of sports facilities	No	2830	76
	Yes	896	24

**Table 2:** Odds ratios for self-reported exercise benefits according to sports facility usage.

	Improvement in Health and Fitness		Stress Relief and Psychological Stability		Vitality and Motivation in Daily Life		Medical Cost Savings		Happiness from Exercise Participation	
	OR (95% CI)	*p*-Value	OR (95% CI)	*p*-Value	OR (95% CI)	*p*-Value	OR (95% CI)	*p*-Value	OR (95% CI)	*p*-Value
Crude	1.31 (1.07–1.62)	0.011	1.38 (1.11–1.71)	0.004	1.62 (1.31–2)	<0.001	1.08 (0.93–1.27)	0.323	1.32 (1.12–1.57)	0.001
Model 1	1.31 (1.06–1.62)	0.012	1.38 (1.1–1.71)	0.004	1.6 (1.29–1.98)	<0.001	1.09 (0.93–1.28)	0.268	1.29 (1.09–1.53)	0.003
Model 2	1.28 (1.04–1.58)	0.022	1.37 (1.1–1.71)	0.005	1.56 (1.25–1.93)	<0.001	1.08 (0.92–1.27)	0.336	1.25 (1.05–1.49)	0.011
Model 3	1.25 (1.01–1.54)	0.043	1.34 (1.07–1.67)	0.01	1.54 (1.24–1.91)	<0.001	1.07 (0.91–1.26)	0.403	1.23 (1.04–1.47)	0.019

The data are represented as odds ratios (ORs) with 95% confidence intervals (CIs). Model 1 was adjusted for sex and age (10-year intervals). Model 2 was further adjusted for types of disabilities and occupation. Model 3 included additional adjustments for living regions and household income. The non-user group was set as the reference category for calculating the OR with the 95% CI.

## Data Availability

The anonymized raw data used in this study are publicly available through the Micro-Data Integrated Services (MDISs) provided by Statistics Korea, a government organization specializing in national statistics (https://mdis.kostat.go.kr, accessed on 9 June 2022). The data supporting the findings of this study can be obtained from the corresponding author upon reasonable request.
